# Perceptions of Resilience and Coping Among Gender-Diverse Individuals Using Photography

**DOI:** 10.1089/trgh.2019.0015

**Published:** 2019-08-28

**Authors:** Jessamyn Bowling, Victoria Schoebel, Chloe Vercruysse

**Affiliations:** Department of Public Health Sciences, UNC Charlotte, Charlotte, North Carolina.

**Keywords:** gender diverse, LGBT, photo-elicitation interviews, resilience, transgender

## Abstract

Gender-diverse individuals (those whose gender and/or sex differ from societal expectations, such as trans or genderqueer) face stigma and discrimination, which can translate into negative health outcomes. Resilience describes the process of overcoming adversity that can mitigate these negative effects. Previous work has focused on trans individuals only or measured resilience without first exploring subjective understandings.

**Purpose:** We sought to explore subjective perceptions of resilience among gender-diverse individuals.

**Methods:** This qualitative study uses longitudinal photo-elicited interviews (in which participants' photos prompt interview discussion) with 21 gender diverse individuals (35 total interviews or a 66% retention rate). Interviews were separated by 6 months and transcripts were analyzed using Dedoose software, with each coded twice by separate coders.

**Results:** Participants' strategies to foster resilience included proactive approaches at both the internal and external levels and distracting/temporary approaches. Two themes, flexibility and awareness, emerged as central facets of resilience trajectories. Flexibility took several forms and was intrinsic to cognitive processes, as well as coping choices, and sought through mental training techniques, such as meditation. Participants demonstrated high levels of awareness by incorporating trauma and health outcomes in gender-diverse populations within their narratives, and through the recognition of the unhelpful aspect of avoidant strategies in building resilience.

**Conclusions:** The diverse ways that individuals perceive their own resilience have implications for resilience research in this group and have the potential to inform the development of strength-based interventions tailored to gender-diverse individuals. Public health interventions targeting resilience have the potential to break the pathway linking adversity to ill health among gender-diverse individuals, thereby reducing health disparities in this group.

## Introduction

Stress can be defined as relationships linking an individual to the environment that are perceived as extremely taxing of one's resources, thereby compromising health.^1^ Minority Stress Model^2,3^ describes how the stress of minoritization translates into negative health outcomes. Gender-diverse individuals, as a minority population, face discrimination and marginalization within the society that put their health at risk.^2,4–6^ This relationship is observed through higher rates of negative mental health outcomes in this population, compared with cisgender individuals,^*^ including anxiety, depression, and suicidality.^4,7,8^ Transgender individuals at present account for approximately one million of U.S. adults.^9^ Research suggests that 39% of these individuals experience psychological distress, nearly eight times the rate of the U.S. population (5%).^8^ Lifetime suicidal ideation affects 82% of transgender adults, against a cross-national prevalence of 9%.^8,10^

Despite these obstacles, gender-diverse individuals may be utilizing strategies to adapt, enhance positive outcomes, and even grow from adversity. Although definitions of the concept vary,^11^ a significant body of research demonstrates that resilience is inextricably bound with stress experiences, and is “a pattern of positive adaptation in the context of past or present adversity.”^12,13^ Resilience, as a nonstatic and multidimensional process, is never absent and may be fostered through various strategies.^14,15^ One such strategy is cognitive flexibility; this may include “(i) adapting to fluctuating situational demands, (ii) reconfiguring mental resources, and (iii) shifting perspective, and (iv) balancing competing desires, needs, and life domains.”^16^ Using resilience frameworks moves beyond a narrow focus on negative health outcomes for gender-diverse individuals to understand resilience trajectories and their underlying mechanisms.^13,17^

Coping, like resilience, is by definition related to stress.^1^ However, the concept differs from resilience in that it refers to the specific strategies, through which demands appraised as taxing within the person–environment relationship are managed either through problem-solving or via emotional regulation.^1^ This is important because repeated stress exposure heightens stress sensitivity.^14^ Adaptation, or resilience, is not only solely reflected by an individual's coping repertoire but also by the ability to sustainably make efforts, mainly cognitive and behavioral, to appropriately respond to stressful demands based on resources.^1^ Myriads of internal and external factors, including health, cultural values, social support, problem-solving skills, or material resources, moderate the transactions intrinsic to the pathway from adversity to resilience.

Although research continues to determine specific factors associated with resilience, such as optimism,^18^ this study focuses on perceived resilience. In this way, we incorporate an emic perspective to identify the ways that participants describe their pathways to resilience, rather than attempting to dichotomize resiliency through the use of scales and statistical analyses. Building from the phenomenological work of Singh et al.,^19^ we examine gender-diverse individuals' perspectives on resilience using photography. An iterative process that examines subjective understandings of resilience may assist in better resilience assessment and interventions to build identified strengths.

## Methods

We conducted longitudinal photo-elicitation interviews (in which interview discussion is prompted by participants' photographs)^20^ with *N*=21 individuals. Two interviews were separated by 6 months (a total of 35 interviews were conducted across the 21 individuals, with 66% of participants completing the second interview^†^), and participants submitted photographs online through Qualtrics for the first interview. We chose to incorporate photography due to the somewhat abstract nature of the concept of resilience, in that photo-elicitation interviewing can help participants think through and materialize abstract concepts in advance of the interview.^21–23^

All participants were at least 18 years old, identified other than cisgender, and lived in Mecklenburg County, North Carolina. Recruitment was done through local lesbian, gay, bisexual, and trans (LGBT) organizations, snowball sampling, and social media advertisements focused on those interested in LGBT issues. Both interviews were conducted in person, and participants gave verbal consent for the interview process and publication of their photographs. For the first interview, participants were asked to submit five digital photographs representing “How are you strong (or not) and what does strength look like?” Although resilience and strength are not synonymous, this term was more encompassing for participants.

Participants were briefed on the photograph guidelines and photography tips (e.g., use natural light if possible, exploring different angles) 1 week before their first interview via phone. Photograph guidelines included the following: (i) must be participant's original photograph and (ii) no identifiable information included (e.g., faces). Their photographs were displayed on a screen during the interview. Furthermore, the interview discussion explored how strength was related (or not) to adversity to explore resiliency. Participants selected their pseudonyms and their pronouns, which are used in this article.

Interviews were audio recorded and transcribed verbatim. We then conducted inductive thematic analyses, in which common concepts are grouped together to form themes.^24^ Analyses during data collection were conducted using Dedoose online software^25^ by a team of three coders, such that each interview was coded twice. A codebook was first established based on the interview guide and augmented after coding the first two interviews. Coders confirmed reliability using Dedoose's test function, with code labels and descriptions revised after discussion for any codes with a kappa of <0.80.

## Results

See Table 1 for participant demographics. Eleven participants (52%) reported their overall health as good or stable, while four participants (19%) described poor health, and seven (33%) described their overall health as both good and bad (often differentiating between mental and physical health). Regarding access to health care, 14 participants (67%) reported utilizing local mental health care either in the past or at present, six of whom (43%) had reported their overall health as good or stable, and felt satisfied with their experiences.

**Table 1.** Photo-Elicitation Interview Participant Demographics (*N*=21)

Pseudonym	Age^a^	Race/ethnicity	Gender identity	Sexual identity
Lara^b^	32	Latinx	Trans woman	Pansexual
Aurora^b^	27	White	Trans woman	Lesbian
Dawn	20	Latina	Trans woman	Bisexual
Espn^b^	32	White	Trans woman	Lesbian
Lynette^b^	28	White	Trans woman	Lesbian
Amy	25	White	Trans woman	Pansexual
Flynn^b^	68	White	Trans woman/strongly female	Bisexual
Natasha	43	White	Male to female trans	Bisexual
Siobhan^b^	62	White	Female with an extra part	Lesbian
Samara	19	White	Trans femme	Sexually fluid
Elijah	30	White	Trans man	Bisexual
Lake^b^	26	White	Trans masculine	Queer
Toby^b^	19	White	Male that happens to be trans	No label
Hava^b^	34	Mixed	Gender queer	Queer
BioDoc^b^	42	White	Gender queer	Pansexual
T	18	White	Gender nonbinary	Pansexual
Nicole	20	African American	Gender nonbinary	Queer
AshWilliams^b^	24	African American	Gender nonbinary	Pansexual
Ciara^b^	24	African American	Gender nonbinary	Likes vaginas
Joy^b^	41	White	Gender nonbinary	Pansexual
E^b^	29	White	Everything	Queer

^a^ Age during first interview.

^b^ Participants completed two interviews.

Out of the 21 participants interviewed, 17 (81%) reported experiencing psychological distress either in the past or at present. Each participant expressed the use of multiple strategies to cope with adversity related to discrimination based on their gender expression or identity (in daily life, in health care, and workplace environments, from family members), body and self-image concerns, and barriers in employment as well as the health care and legal systems. Strategies evolved, depending on adversity type, stress load, resilience development, and available resources.

### Proactive strategies: internal

Participants described proactive strategies that were at the intrapersonal level. These included reframing, boundary setting, flexibility, meditation, and making adequate coping choice.

Participants described several strategies in re-evaluation or contextualization of their adversity. One aspect of this was to “own it” (meaning have confidence in oneself) or work with the situation or body they were living in (Table 2[1(A)]). Elijah tried cultivating positivity despite the struggles associated with being gender diverse, including bathroom-related barriers (Table 2[1B]). These are examples in which participants re-evaluated negative mind-sets regarding their appearance to prevent intrusive thoughts and cultivate confidence. This proactive strategy helped them mitigate internalizing discrimination and narrow physical ideals.

**Table 2.** Themes of Resilience Among Gender-Diverse Individuals with Example Quotations

Type	Category	Example quotation
1. Proactive: Internal	Reframing	(A) “If I feel uncomfortable [with my appearance], everyone is uncomfortable. So I own it.” (Siobhan)
		(B) “Just the fact that it's a debate whether or not I'm allowed to use the bathroom, it takes strength to deal with that every day. So just being part of the community and being who I am takes strength. And I think that remaining proud of that, I am proud of it, I am happy with who I am and I don't hide it… I think it's a choice… Being okay with myself takes strength…I wish the T [testosterone] made me hairier, my voice was deeper… It takes a lot of resiliency to not focus on that and not think about how I don't pass all the time.” (Elijah)
	Reframing: Contextualizing the struggle	(C) “I think one of my favorite coping mechanisms is just the idea that today will end and tomorrow will begin… I've found that the worst days aren't as bad as they have been or could have been. You just have those days where you just get home and you're putting spinach in a pot, you're just eating it and you're angry, you're just kind of kaput. Then I look outside and the sun's going down and I'm like ‘But it's over.'” (Nicole)
		(D) “Our entire vessel is scar tissue… Our bodies have solely been reactionary things, since we were born… We're just kind of like walking, healing vessels, whether it's our emotions. And so I think, especially in queer community… we have to acknowledge trauma, and move in a very trauma-informed way.” (Ciara)
	Meditation	(E) “It's one thing that actually keeps me grounded, centered, a moral compass.” (Natasha)
	Boundary setting: Work	(F) “It's hard to learn where my boundaries are and stick to them because it can hurt sometimes… And to make sure I have enough self-awareness to know when I'm setting a boundary or sticking to a boundary because of [work] and know when it's because I'm frustrated at that person or the situation.” (Elijah)
	Boundary setting: Personal	(G) “I'm not gonna let whatever insecurity you haven't dealt with in your life be placed on my personhood.” (Ciara)
		(H) “[During my transition] I had seen some people I'm not close to at work, and they want to be supportive in some way, but more than that, I almost feel like they want to be a part of the experience [of transitioning]. I get that they're supportive of LGBT people, and maybe they want to show that support for me. But we don't have that relationship, and I don't need their support. I'm not trying to be mean at all, but there's no support for you here. I don't need that.” (Lynette)
	Choice of coping strategy	(I) “Coping is doing the best you can, the healthiest coping mechanisms you can grasp, any given day. Which some days means sitting on the couch and eating ice cream and watching TV.” (E)
2. Proactive: External	Social support	(A) “That's what makes me feel strong too, is when my friends stick together and help each other out… No matter if my friend doesn't have food, he'll give food to the next person. I'm totally fine.” (T)
		(B) “I'm relying on my chosen family a lot to process things and… it's almost like we're each other's therapists.” (AshWilliams)
	Advocacy	(C) “The way that I organize right now is not sustainable… It feels like a really big deal to me, like this is the most important thing that I think I'm doing right now or I've done for a long time… Maybe we're not talking about it [social justice issues] the right way, or maybe people feel polarized if we say they don't like transfemmes who are black. Maybe that is not going to make someone sit down and talk to us about this.” (AshWilliams)
	Hobbies	(D) “In terms of racial, cultural identity and my fluctuating and sexuality [music] helped me feel seen.” (Hava)
		(E) “I basically skate alone and listen to music. It kind of revives me. I really take pride in the energy that it brings” (T)
	Faith	(F) “I have to do a lot of praying, self-introspection, reinforcement…Sometimes I'll go to the greenway just so I can be outside, be in the created world, and open myself to feel closer to God and let God kinda take it away from me and reaffirm to me that, “That's okay, you did what you could.” (Flynn)
	Food	(G) “You have to eat right, and drink right, and you can't load yourself up on sugar or caffeine and garbage, that's just prolonging your crash” (Espn)
3. Distracting/Temporary	Reading	(A) “Reading is a great escape. It's good for building your vocabulary and leaving this world and going to another one.” (Aurora)
	Ignoring issues	(B) “It's kind of like riding a bicycle through a neighborhood where nobody locks their dogs up and they are all pit bulls and rottweilers… You just have to keep driving as fast as you can through that neighborhood on that bike because if you slow down, either the dogs would notice you or they will catch you, and I mean it's bad news… Every situation can be dangerous. So I just ignore it.” (Lara)
	Violence preparedness	(C) “Trans women actually need to be prepared to murder their intimate partners at any time in case they feel so ashamed that they try and turn on us.” (Dawn)
4. Avoidant	Substance use	(A) “That's the result of self-harm a couple of years ago… I used to drink a lot, and I've done my fair share of drugs… this picture and the alcohol consumption relate to a period of extreme denial cause at the time I couldn't tell you why I was doing that… Probably once I really understood [that I was trans] and that I was OK with that, once the denial ended, I didn't have any compulsion to do things like that anymore.” (Amy)
	Anger	(B) “I'm just quick to anger… We were in line at the grocery store… I was paying, and the guy next to me was standing right next to me, and I said, “Hey, could you not stand so close? I'm not done yet.” And the guy was like, “Oh, I'm just on my phone,” and I was getting ready to yell at the guy…And I just was like, “Just go zen. Just go fucking zen. Don't.” So I just turned my back to the guy and physically-Whereas in the past, I would've been like, “Get the fuck out of my space,” so still, it's there. I was really proud of myself.” (Hava)

LGBT, lesbian, gay, bisexual, and trans.

**FIG. 1.**
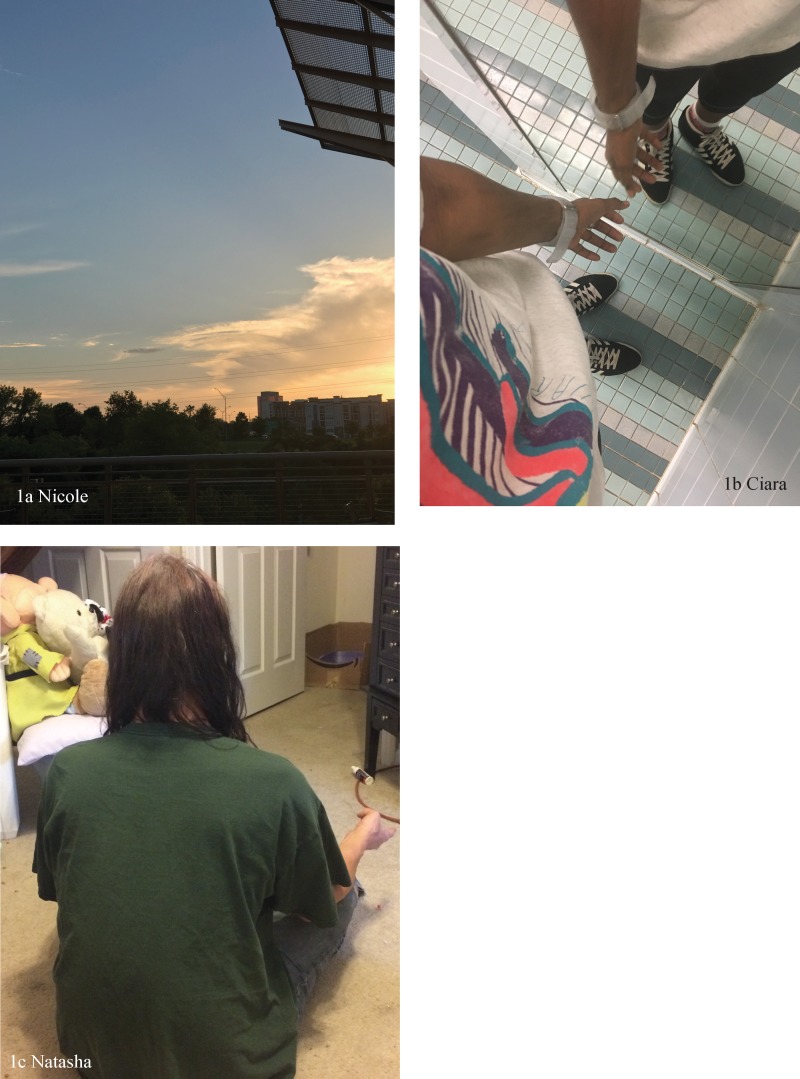
Participants' photographs related to proactive internal strategies.

Second, participants contextualized specific struggles by saying that they would eventually end or there would be a respite from the hardship. Nicole described their specific reframing of a long or stressful day as a finite period of time (Table 2[1C], see Fig. 1a for their photograph of the sunset). Another cognitive process that was important for resilience included the incorporation of trauma into individual narratives. Toby acknowledged statistics for trans people in situating their overall progress in life, concluding that graduating high school and even getting out of bed were achievements. Similarly, Joy acknowledged the people in her life who passed because of mental health issues and her gratitude to have survived. Ciara's photograph (Fig. 1b) points to what they say is “the reflective nature of scarring” (for more of a description, see Table 2[1D]).

Setting boundaries in the workplace and in personal lives was described as resilience-enhancing. Elijah refers to setting boundaries at work in resource allocation to clients to prevent taking out their stress from work-related discrimination on clients, such as denying clients extra resources when requested (Table 2[1F]). Ciara describes family boundaries around their gender expression and marijuana use (Table 2[1G]). Lynette's boundaries included directly addressing things before they progressed; for example, she refused coworkers support during her physical transitioning, told potential partners about her gender identity early, took the initiative to call her insurance company to ask about voice therapy coverage, and addressed ignorant comments directly (Table 2[1H]). Participants expressed pride in these strategies because they were learned over time and took effort to carry out.

Flexibility was a key element of many participants' strategies, both in their mind-sets and choosing their coping strategies. All types of cognitive flexibility were described by participants, although shifting perspective was described most in-depth. Meditation was important for some participants in building self-awareness and “grounding” (Table 2[1E], see Fig. 1c for Natasha's photograph of meditating). Choosing health beneficial coping strategies over detrimental ones was a source of resilience for some participants. Less healthy choices were framed as temporary (Table 2[1I]).

### Proactive strategies: external

Participants also identified strategies that helped to proactively mitigate adversity that was external to them. These included social support, advocacy, hobbies, faith, and food.

**FIG. 2.**
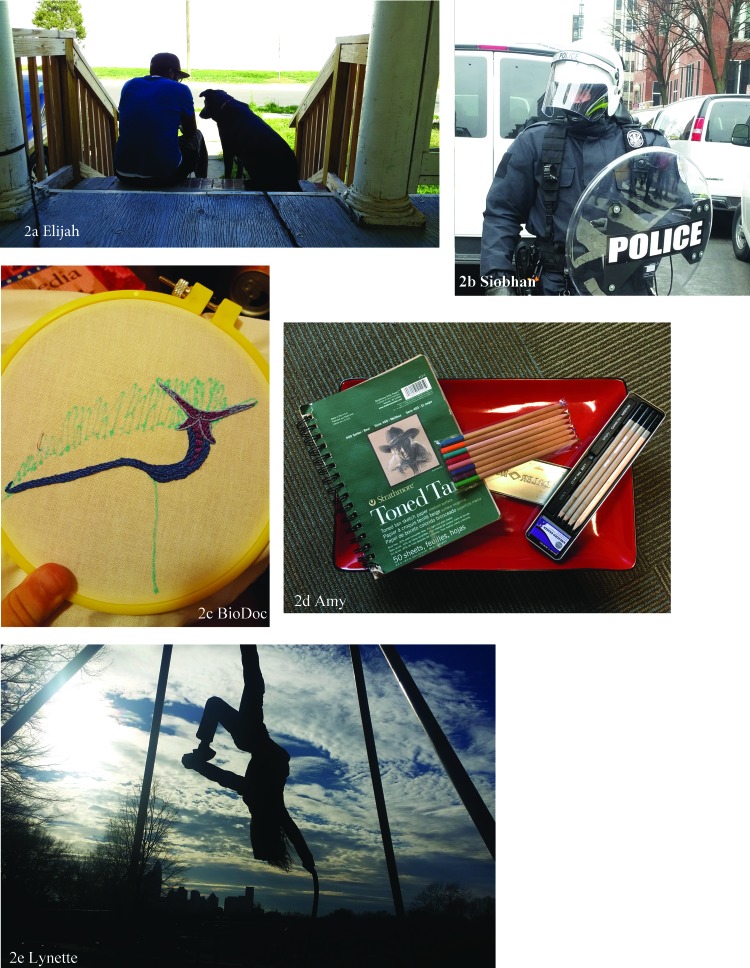
Participants' photographs related to proactive external strategies.

Social support facilitated resilience directly through sharing of resources and strategies to navigate adversity. T was friends with individuals experiencing homelessness and valued their generosity (Table 2[2A]). Social support also buffered adversity through emotional support, as AshWilliams described it as therapeutic (Table 2[2B]). Elijah included a photograph of his partner and dog to reference the emotional support that he gets from both these sources (Fig. 2a). Pets were an important aspect of emotional support, as a constant nonjudgmental presence in participants' lives.

Twelve participants described their advocacy efforts in building resilience by counteracting the social identity threats intrinsic to the experience of being trans and nonbinary. This included formal protests and organizing, as well as more casual conversation, and spanned across social justice issues (gender and sexual identities, race/ethnicity, immigration status, disability status). Siobhan included a photograph of police blocking her during a protest (Fig. 2b). For eight participants, advocacy was a way to decrease psychological distress, enact agency, and foster resilience. It gave participants a purpose, helped their community, and helped them stand up for themselves.

Regardless of whether they felt embedded in a community of activists, advocating for themselves and others was an important facet of many participants' resilience. For four participants, however, advocacy was something that negatively influenced or worsened psychological distress, especially in the case of one participant facing felony charges for protests. AshWilliams described a process of re-evaluating their efforts when change was deemed as insufficient for them (Table 2[2C]). This type of re-evaluation likely improves advocacy efforts but is also a never-ending, and consuming, process. Thus, for these participants, acts of advocacy sometimes felt taxing, and put them in stressful and risky situations thereby negatively impacting their physical health and psychological well-being.

Practicing new skills improved self-efficacy, or one's confidence in being able to accomplish tasks. BioDoc discussed the ways they learned new skills that were both traditionally seen as feminine (such as crafting, baking, and gardening) or masculine (smoking meat and brewing beer) (see Fig. 2c for their needlepoint).

Art (both creating it and appreciating it) emerged as a proactive coping mechanism for addressing adversity and stress. Art allowed a space for expression. Participants made and listened to music, wrote fiction, and drew. Amy's notebooks were a way of communicating or, in her words, “expressing myself to myself” (Fig. 2d). Listening to music helped Hava feel recognized in their identities (Table 2[2D]). Exercise was an important strategy for some participants, not only as enhancing physical abilities and strength but also as a mental health support. Lynette worked to get her body strong before beginning hormone therapy, so she could continue aerial arts (Fig. 2e).

**FIG. 3.**
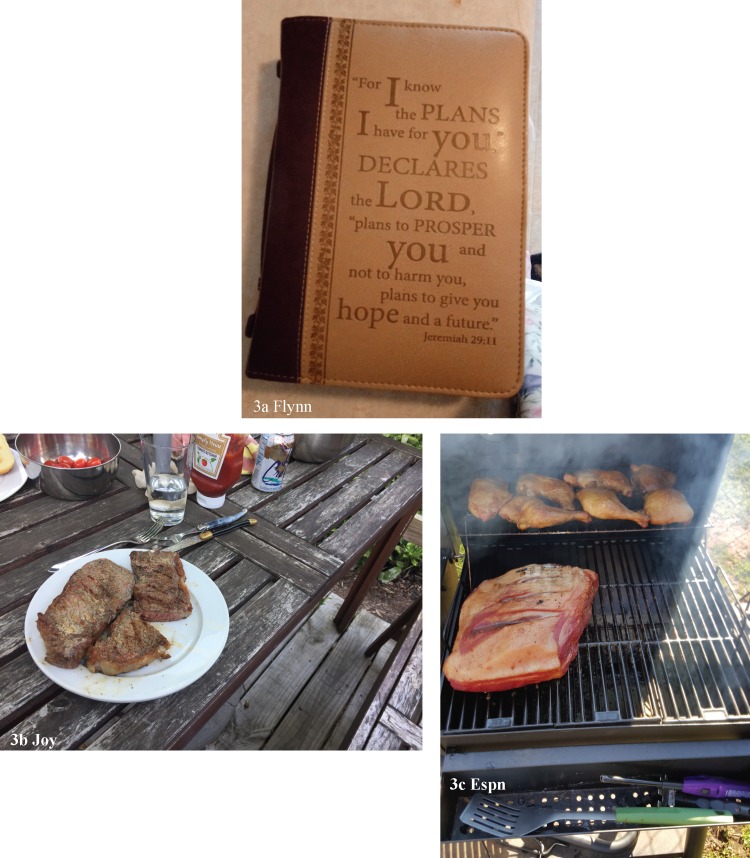
Participants' photographs related to proactive external strategies continued.

E struggled with the enforcement of a gender binarism in Buddhism, yet information online helped them understand how to make sense and respond to this religious stance. Natasha's meditation practice is part of her Wiccan faith. Flynn, a pastor, had previously struggled with reconciling negative Biblical texts related to her gender. After reinterpreting them, her faith in God pulled her through many difficult situations (Table 2[2F]; see Fig. 3a for her bible cover).

Eating in a way that was nourishing and cooking supported participants' physical and mental health. For some, cooking represented their self-efficacy and goal fulfillment. Nicole was proud of their present eating habits because after moving around a lot and after her parents' passing she had finally found her own rhythm. Joy similarly had gone through a bad divorce and raising two small children in which she struggled to regain stability. Cooking for her family was a part of this stability (see Fig. 3b for her grilled dinner). For others, it was a “time filler” that helped distract from stress or loneliness in a positive way. Espn, a truck driver, cooked food for others for socializing and health purposes (Table 2[2G]; see Fig. 3c). Participants described times of stress alongside not being able to eat or plan meals as they wanted, thereby depicting food as an indicator of overall well-being.

### Distracting/temporary

**FIG. 4.**
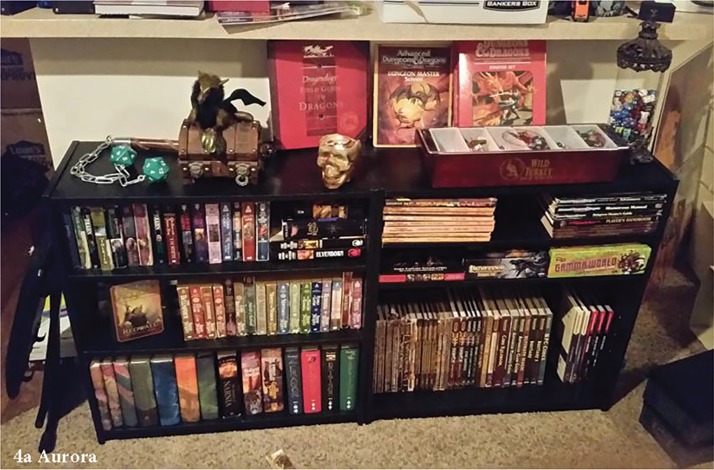
Participants' photographs related to distracting or temporary strategies.

Participants described some coping strategies that could offer relief from adversity but did not necessarily contribute toward addressing the source of their stress. Participants used art, TV, and books as an escape, in part, because they were a constant support. Aurora relied on reading to escape and saw it as a benefit (Table 2[3A]; see Fig. 4a). E also described ignoring adversity, which helped them to reduce immediate stress when facing discrimination. Another participant compared adversity to dangerous dogs while bicycling through a neighborhood: you had to bike quickly, so the dogs didn't notice you or catch up to you (Table 2[3B]). Dawn spoke about violence preparedness as a strategy that is specific to the risks associated with intimate life among trans women and aimed to ensure survival (Table 2[3C]). Given that this strategy does not reduce the stressor of violence and may result in further issues (in the case of enacting violence on a partner), this is a temporary strategy.

### Avoidance

**FIG. 5.**
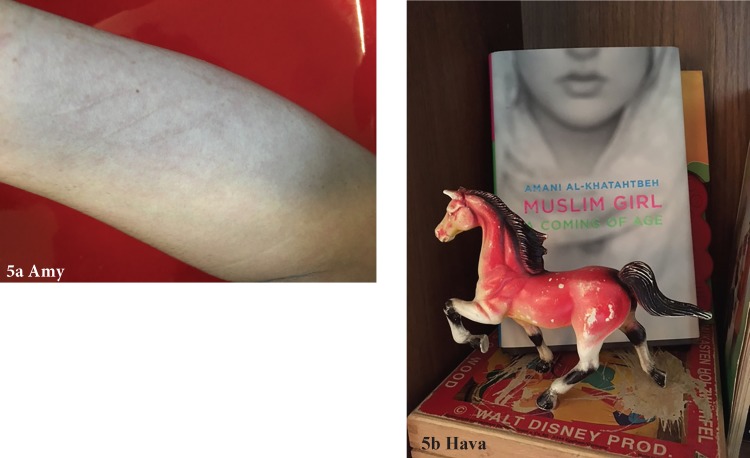
Participants' photographs related to avoidance.

Notably, nonadaptive (long-term resilience development) coping strategies emerged from the participants' narratives. Many participants described previous as well as present use of alcohol and drugs to cope with adversity. Referencing Figure 5a, Amy explains how the identification of her gender identity was the trigger that prompted her to stop engaging in self-harming behaviors (Table 2[4A]). Hava described their previous use of substances connected to the anger that they have since started managing through physical activity (Table 2[4B]; see Fig. 5b for their childhood toy of a horse in a confrontational stance). Although Siobhan still uses marijuana and alcohol (although considerably less than her previous cocaine and meth use), none of the participants described their substance use as a resilience-building strategy.

## Discussion

This article describes gender-diverse individuals' perceptions of their own resilience. Many of these may not be unique to gender-diverse individuals, and it is important to identify the universalities as well as the specifics. The participants specifically framed advocacy, violence preparedness, and substance use strategies within the context of their gender-diverse identities. Several themes overlap with previous resilience work with gender-diverse individuals. Singh et al.^19^ identified awareness of oppression and connection with a supportive community, which shares similarities with our finding of incorporation of trauma or oppression into one's mind-set and social support.

Participants described both internal and external aspects of resilience. One prominent external theme was social support. Social support supported resilience through buffering, specifically the “extremes of anxiety and loneliness.”^26^ However, social support also supported resilience directly through learning from others' strategies in maintaining health and positive emotions. This echoes Pinto et al.,^27^ in which trans individuals' social networks provided medical resources and political advocacy.

Previous literature has described how social support can facilitate hobbies^28^ and reduce depression and anxiety among gender-diverse individuals.^29^ Here, hobbies were generally framed as separate from social interactions, in that they were opportunities for participants to practice new skills and express aspects of their personalities.

Existing research among other marginalized populations shows that social support may have a negative impact on health depending on the composition of the social network it derives from.^30^ Similarly, in the present context, the resilience enhancing effect of social support varied as a result of the stigma inherent to the experience of being trans and nonbinary. Ending relationships that were negative or discriminatory limited social support, but reduced adversity for participants. Notably, although closing off from others generally reduced exposure to stigma, it did not offer relief from related negative emotions. The role of advocacy in our study resembles Singh and McKleroy's^31^ identified theme of connections with the activist community as supporting resilience; however, participants in our study described the act of advocacy itself as building resilience for them.

Avoidant coping mechanisms have been associated with depression and anxiety for gender-diverse individuals, yet substance use is a common coping mechanism in this population.^32–35^ Budge et al.^28^ found that as transgender individuals advanced in their transition process, they were less likely to use avoidant coping mechanisms. This theme resonates with one of our participants' (Amy) resilience narrative and suggests a need for more nuanced and longitudinal examinations of resilience among gender-diverse individuals.

### Implications and future directions

Food, including eating and food preparation, reinforced self-efficacy and distracted from loneliness. Food's relationship to resilience in academic literature is mostly limited to reducing or preventing disordered behaviors.^36, 37^ Higher rates of disordered eating and body image issues have been documented for transgender individuals.^38,39^ However, research is lacking to understand the complex pathways through which food connects to gender-diverse individuals' resilience.

Another external element that a few participants highlighted was the role of faith and faith communities in their resiliency. Previous research has explored how Christianity can provide stigmatizing messages about a transgender identity.^40,41^ However, more research is needed to explore other faiths, including Buddhism, and other gender-diverse identities in relation to spirituality and resilience.

For practitioners working with gender-diverse individuals, participant photography is a successful way of prompting reflection on resilience. Approaching resilience as a dynamic process, versus “a static unitary property of the person,” underlines the changeable nature of the concept, thereby unfolding opportunities for intervention.^14^ Although coping strategies supporting resilience should be targeted, it may be important to interrogate first about the nuanced health influences for these strategies. Participants in this study, for example, reported both positive and negative influences of their social connections and advocacy efforts.

Furthermore, coping strategies may counteract one another. For example, participants described strategies that may have been calming but limited their social support or direct dealing with situations (such as reading to escape). Given the central aspect of cognitive flexibility in our findings, and to understand the process of coping flexibility for gender-diverse individuals, longitudinal research should be undertaken to inform intervention development. This study also has implications for research operationalization and measurement of resilience among gender-diverse individuals. In measuring resilience quantitatively, existing resilience scales may not capture the various ways gender-diverse individuals view their resilience.

### Strengths and limitations

This study addressed gender-diverse individuals by including nearly half of participants who identified with a category that would not be encompassed within “trans” (e.g., gender nonbinary, bi gender). This study used photo-elicitation interviews, which gave the participants a unique space to freely and creatively highlight their individual lived experiences.

Although this study has various strengths, there are some limitations. There were few photographs representing the internal aspects of resilience, which may be due to the difficulty of representing this in visual form; there were also few photographs of the distracting/temporary and avoidant types of coping strategies, potentially due to social desirability biases. The majority of participants were younger than 40 years, and our findings may be more applicable for this segment of the population. Most of the participants in this study identify as white/Caucasian, which obscures the unique experiences of racial or ethnic minorities. The research team was predominantly cisgender identified but attempted to mitigate the impact of this by incorporating community-based approaches.

## Conclusion

Most of the participants experienced psychological distress but deliberately used multiple coping strategies, both proactive and passive, to mitigate the effects of adversity, foster resilience, and enhance mental health outcomes. The participants' narratives demonstrate the complexity of resilience trajectories among gender-diverse individuals where unique strategies may both induce and hinder resilience simultaneously. Participants are actively navigating and reflecting on their individual resilience, while being aware of negative outcomes for gender-diverse populations.

## Notes

^*^ For the purposes of this study, gender-diverse individual refers to any individual with a non-normative gender based on societal expectations of their sex assigned at birth, which includes, but is not limited to, individuals who are trans, genderqueer, nonbinary, or gender fluid. Conversely, cisgender individuals are those whose gender corresponds to their sex assigned at birth.^5^ For an overview of how individuals may use gender-diverse terminology and how it relates to health, see Budge et al.^7^

^†^ Participants received three e-mail and text reminders, with the last reminder 6 weeks after the target date. There were no discernible trends among participants not completing the second interview based on age, race/ethnicity, or gender identity.
